# Copy number loss of (src homology 2 domain containing)-transforming protein 2 (*SHC2*) gene: discordant loss in monozygotic twins and frequent loss in patients with multiple system atrophy

**DOI:** 10.1186/1756-6606-4-24

**Published:** 2011-06-10

**Authors:** Hidenao Sasaki, Mitsuru Emi, Hiroshi Iijima, Noriko Ito, Hidenori Sato, Ichiro Yabe, Takeo Kato, Jun Utsumi, Kenichi Matsubara

**Affiliations:** 1 Department of Neurology, Graduate School of Medicine, Hokkaido University, North 15, West 7, Kita-ku, Sapporo 060-8638, Japan; 2 CNV Laboratory, DNA Chip Research Institute, 1-1-43 Suehirocho, Tsurumi-ku, Yokohama, Kanagawa 230-0045, Japan; 3 Department of Neurology, Haematology, Metabolism, Endocrinology, and Diabetology, Yamagata University Faculty of Medicine, 2-2-2 Iida-Nishi, Yamagata, Yamagata 990-9585, Japan; 4 Creative Research Institution, Hokkaido University, North 21, West 10, Kita-ku, Sapporo 001-0021, Japan; 5 Graduate School of Pharmaceutical Sciences, Kyoto University, 46-29 Yoshida-Shimo-Adachi-cho, Sakyo-ku, Kyoto 606-8501, Japan

**Keywords:** Multiple system atrophy, copy number variation, phenotypically discordant monozygotic twins, (Src homology 2 domain containing)-transforming protein 2, subtelomere, ataxia, parkinsonism, disease-susceptibility gene

## Abstract

**Background:**

Multiple system atrophy (MSA) is a sporadic disease. Its pathogenesis may involve multiple genetic and nongenetic factors, but its etiology remains largely unknown. We hypothesized that the genome of a patient with MSA would demonstrate copy number variations (CNVs) in the genes or genomic regions of interest. To identify genomic alterations increasing the risk for MSA, we examined a pair of monozygotic (MZ) twins discordant for the MSA phenotype and 32 patients with MSA.

**Results:**

By whole-genome CNV analysis using a combination of CNV beadchip and comparative genomic hybridization (CGH)-based CNV microarrays followed by region-targeting, high-density, custom-made oligonucleotide tiling microarray analysis, we identified disease-specific copy number loss of the (Src homology 2 domain containing)-transforming protein 2 (*SHC2*) gene in the distal 350-kb subtelomeric region of 19p13.3 in the affected MZ twin and 10 of the 31 patients with MSA but not in 2 independent control populations (*p *= 1.04 × 10^-8^, odds ratio = 89.8, Pearson's chi-square test).

**Conclusions:**

Copy number loss of *SHC2 *strongly indicates a causal link to MSA. CNV analysis of phenotypically discordant MZ twins is a powerful tool for identifying disease-predisposing loci. Our results would enable the identification of novel diagnostic measure, therapeutic targets and better understanding of the etiology of MSA.

## Background

Multiple system atrophy (MSA; MIM146500) is a progressive neurodegenerative disease clinically characterized by a variable combination of cerebellar ataxia, autonomic disturbance, and parkinsonism with a poor response to levodopa. X-ray computed tomography or magnetic resonance imaging (MRI) studies of the brain usually detect atrophy of the cerebellum and brain stem; MRI also reveals abnormal signal intensity in the white matter of these structures and in the putamen. The neuropathologic features of MSA are neuronal loss, astrogliosis, and argyrophilic glial cytoplasmic inclusions (GCIs) in oligodendrocytes [1]. GCIs involve the aggregation of insoluble fibrillar α-synuclein [2], and the *SYNA *locus has been associated with MSA in some genetic studies [3,4], but not in others [5]. The neuroimaging findings, clinical features, and neuropathologic features constitute the current diagnostic criteria for MSA [6]. MSA essentially is a sporadic disorder with onset in adulthood. The involvement of environmental factors and epigenetic mechanisms in its pathogenesis has been postulated; however, its etiology remains largely unknown.

Recently, copy number variations (CNVs) have been recognized as important interindividual structural variations often located in the complex repetitive regions of the human genome. They account for more nucleotide variations between individuals than single-nucleotide polymorphisms (SNPs). Recent studies have indicated that CNVs are considerable contributors to genomic diseases and disease susceptibility in humans [7,8]. In addition, researchers have developed a molecular strategy that takes advantage of the unique genetic characteristics of monozygotic (MZ) twins: they predicted that analysis of MZ twins discordant for a given disorder (i.e., one is affected and the other is not) will provide important clues for identifying the underlying genetic mechanism of that disorder [9,10]. Because a genetic difference between MZ twins is an extreme form of somatic mosaicism, de novo CNVs observed in the affected co-twin is a potential characteristic of the CNV region harboring the disease-susceptibility gene(s).

**Figure 1:**
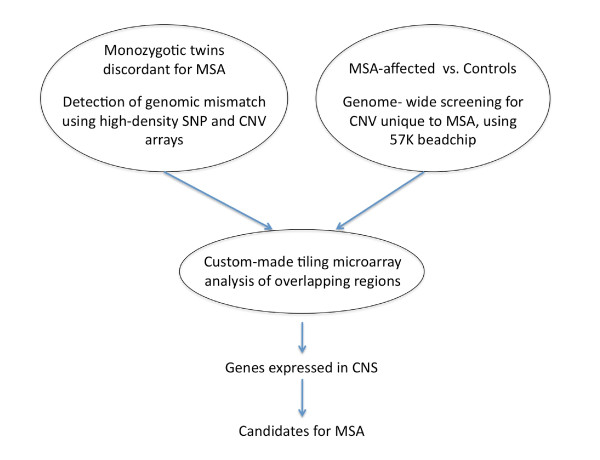
**Strategy for identifying genes susceptible to MSA**.

Accordingly, we hypothesized that the genome of a patient with MSA would demonstrate CNVs in the genes or genomic regions of interest. To test this hypothesis, we examined whether a pair of MZ twins discordant for the MSA phenotype display differences in genomic structure as de novo CNVs and searched for CNVs in the genomes of 33 patients with MSA, including MZ twins. We conducted whole-genome CNV analysis by using a genome-wide oligonucleotide CNV microarray and CNV beadchips, both of which focus on the CNV-rich region of the human genome. To validate the results of the whole-genome analysis, we characterized the genetic alteration by using a region-targeting, high-density, custom-made oligonucleotide tiling microarray. Our research strategy is illustrated in Figure 1. Throughout these procedures, we searched for CNVs rare in normal individuals but frequent in patients with MSA. A gene whose transcription is affected by such CNVs and whose expression is mostly confined to the brain must be a crucial candidate for the pathogenesis of MSA.

## Methods

### Subjects

The study comprised 33 unrelated patients with MSA including a pair of MZ twins discordant for the MSA phenotype (Table 1). All the patients were neurologically evaluated at the Department of Neurology, Hokkaido University Hospital, and examined by brain MRI. The mean (SD) age of MSA onset was 58.1 (8.2) years (age range = 37-72 years). According to the current consensus criteria, 31 patients had probable MSA (10 with MSA-P and 21 with MSA-C) and 2 had possible MSA (one with MSA-P and one with MSA-C) [6]. Of the latter 2 patients, the affected MZ twin (aged 67 years) subsequently satisfied the criteria for probable MSA-C, with obvious atrophy of the brainstem and cerebellum on brain MRI. His disorder began at age 59, but his twin was asymptomatic during this study (Table 2).

**Table 1:** Profile of the subjects

Subjects	*N*	Male-to-female ratio	Age at sampling (range; years)
Patients with MSA	33*	18:15	61.5 ± 8.3 (39-77)

First set of controls	100*	53:47	57.4 ± 8.8 (41-76)

Second set of controls	25	17:8	59.3 ± 8.9 (41-76)

*A pair of MZ twins was included in each group.

**Table 2:** Clinical features and phenotypes of the 33 patients

Evaluation	Predominant features	Number (%)
At admission	Parkinsonism	8 (24)

	Cerebellar ataxia > dysautonomia	24 (73)

	Dysautonomia	1 (3)

At sampling	Probable MSA-P	10 (30)

	Possible MSA-P	1 (3)

	Probable MSA-C	21 (64)

	Possible MSA-C	1 (3)

One hundred control subjects were randomly selected from community-dwelling elderly individuals with no neurologic diseases (first set of healthy controls; Table 1) [11]. Fisher's exact test showed no significant differences in the mean age or male-to-female ratio between the MSA-affected patients and the first set of control subjects. In addition, chi-square test showed the absence of significant deviation in distribution between the groups. We extracted DNA from peripheral blood leukocytes but not from lymphoblastoid cell lines.

All the subjects gave written informed consent for genetic analysis. The study was approved by the Medical Ethics Committees of Hokkaido University Graduate School of Medicine and Yamagata University Faculty of Medicine.

### Whole-genome CNV microarray analysis of the MZ twins

After labeling with Cy5 (test) or Cy3 (reference) dye, the DNAs from the MZ twins discordant for the MSA phenotype were competitively hybridized in a human CNV microarray (SurePrint G3 Human CNV 400K Microarray, Agilent Technologies, Santa Clara, CA) and were washed and scanned according to previously described procedures [12]. Further, the DNAs were separately assayed against a single reference sample (NA19000, a HapMap Japanese male). All the hybridizations were performed in duplicate, and the dyes were interchanged between the DNAs in the second hybridization to eliminate a possible dye-specific bias. Moreover, to validate monozygosity with multiple SNP probes, the DNAs were hybridized with CytoSNP-12 beadchips (Illumina, Inc., San Diego, CA) and scanned for the resulting 300,000 SNP calls.

### Whole-genome CNV beadchip analysis of the patients with MSA

We used whole-genome CNV beadchips (57K, i-select format, Illumina Infinium system; deCODE Genetics, Inc., Reykjavik, Iceland) with CNV probes to target the CNV-rich region of the whole genome as previously described [11]. The probe content comprised 15,559 CNV segments covering 190 Mb or 6% of the human genome. The platform has been tested among 4,000 Icelandic and HapMap samples. The test showed that 7,880 of the 42,800 univariant probes called were CNVs with minor allele frequency of > 5%. Over 3,742 CNV segments of size range 5-60 kb were defined; these segments are 18-fold enriched for CNVs compared with the genome-wide average density [13].

### High-density custom-made oligonucleotide tiling microarray analysis

We fabricated a custom-made microarray comprising 60-mer probes (Agilent Technologies) targeting a 350-kb genomic region in the distal subtelomeric region of 19p13.3 (Chr. 19: 250,000-600,000 [NCBI Build 36.1, hg18]) and used the Agilent website [12] to select and design the custom tiling array for DNA analysis of the patients with MSA. The tiling microarray experiments were performed as described previously [11]. For the twin analysis, the DNAs from the MZ twins were competitively hybridized with custom tiling microarrays as described for the whole-genome CNV microarray analysis (CNV 400K microarray).

### Statistical analysis

Data analysis of the microarray experiments was conducted by using the Aberration Detection Method-2 statistical algorithm (Agilent Technologies) on the basis of the combined log_2 _ratios at a threshold of 5 as was done in our previous study[14]. The data were centralized, and calls with average log_2 _ratios of < 0.15 were filtered to exclude false positives.

Data analysis of the CNV beadchip experiments was conducted by using DosageMiner software (deCODE Genetics). The loss/gain analysis consisted of the following 4 steps: (1) intensity normalization and GC content correction, (2) removal of batch effects by using principal component analysis, (3) calling of clusters by using a Gaussian mixture model, and (4) determination of CNV type by using graphical constraints. In brief, CNVs were identified when CNV events were conspicuous among the data, because all sample intensities for CNV probes should increase or decrease relative to those for the neighboring probes (not in the CNV region). To determine deviations in signal intensity, we first normalized the intensities. The normalized intensities for each color channel were determined by using an equation and fit formula (deCODE Genetics). A stretch with more than one marker showing abnormality in copy number compared with a consecutive stretch in the genome is considered more likely evidence of deletion or gain [13]. Statistical analysis of the clinical data was performed with chi-square test or Fisher's exact test in statistical program R [15].

## Results

### Whole-genome CNV array analysis of MZ twins

According to a previous molecular strategy, we investigated a pair of MZ twins discordant for the MSA phenotype [9,10]. We confirmed their monozygosity on the basis of > 99% concordant genotypes by SNP beadchip analysis containing more than 300,000 SNPs.

CGH-based whole-genome CNV oligonucleotide microarray analysis for initial screening of genome alterations in the twins revealed putative variations in each twin. DNA assay against a normal Japanese reference sample (HapMap NA1900) confirmed the true genotype of the twins. We identified the following 3 regions with specific copy number loss in the MSA-affected twin (HK33): a CNV region on 2p25.3 (genomic positions 3,642,547-3,643,266, with 2 probes [16]), a CNV region on 4q35.2 (genomic positions 187,590,335-187,594,679, with 32 probes [16]), and a CNV region on 19p13.3 (genomic positions 249,367-252,260, with 35 probes [16]). The patterns of the 3 CNV regions are displayed in Figure S1 in Additional file 1, Figure S2 in Additional file 2, and Figure S3 in Additional file 3 respectively.

The last region on 19p13.3 is located within the 19p subtelomere (250 kb from the 19p telomere). Because a subtelomere is associated with abundant multicopy repeats or CNVs known as rearrangement hotspots, predisposing individuals to deletion or duplication events and possible pathogenic alterations, we hypothesized that this copy number loss on 19p13.3 predisposes individuals to MSA and thus focused on this region for subsequent analysis.

### Whole-genome CNV beadchip analysis of patients with MSA

We screened 33 patients with sporadic MSA, including the MZ twins, and a set of 100 controls by whole-genome CNV beadchip analysis (Table 1). Of the markers that showed CNVs, 3 genomic regions (i.e., CNV regions on 4p16.3, 20q13.3, and 19p13.3) displayed frequent copy number loss in 10 patients with MSA but not the controls (*p *< 1.0 × 10^-8^).

**Figure 2:**
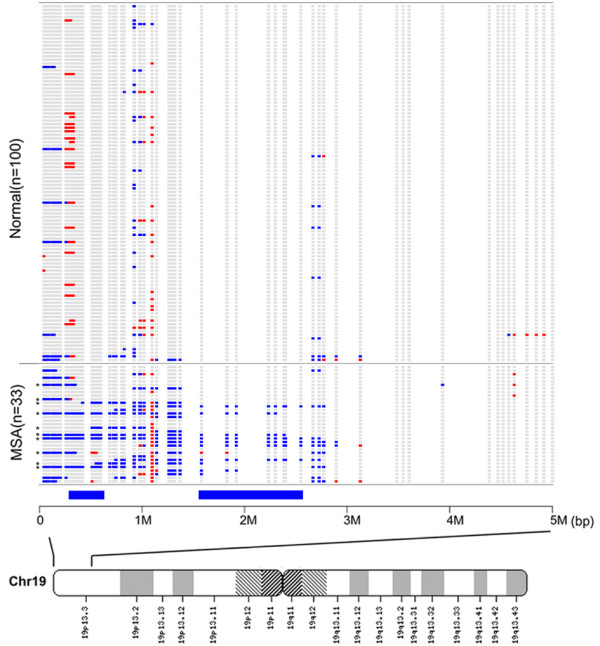
**Genomic region harboring copy number loss on 19p13.3 in patients with MSA**. Data measured by CNV 57K beadchip analysis were analyzed by using the Hidden Malcov Model. The genomic structures of 100 normal control subjects (top) and 33 patients with MSA (bottom) are horizontally aligned from position 000,000 (left) to position 5,000,000 (right). Each blue square represents copy number loss at each CNV probe site whereas each red square represents copy number gain. The horizontal blue bars at the bottom show the distal 350-kb region and proximal 1-Mb region with frequent copy number loss. The asterisks to the bottom left indicate cases of *SHC2 *deletion. The bottom map shows the positions of putative genes in the region [16].

Among the 3 CNV regions with structural differences between the MZ twins, frequent copy number loss was observed on 19p13.3, but not on 2p25.3 (Figure S4 in Additional file 4) and 4q35.2 (Figure S5 in Additional file 5), in 10 patients with MSA compared with the first set of controls (*p *= 1.04 × 10^-8^, odds ratio = 89.8, Pearson's chi-square test; Figure 2). Because the 19p13.3 region (comprising the distal 350-kb region and the proximal 1-Mb region in Figure 2) showed copy number loss in both the affected MZ twin and 10 patients with MSA, we focused on this region for further analysis.

### High-density tiling microarray analysis of the MZ twins

**Figure 3:**
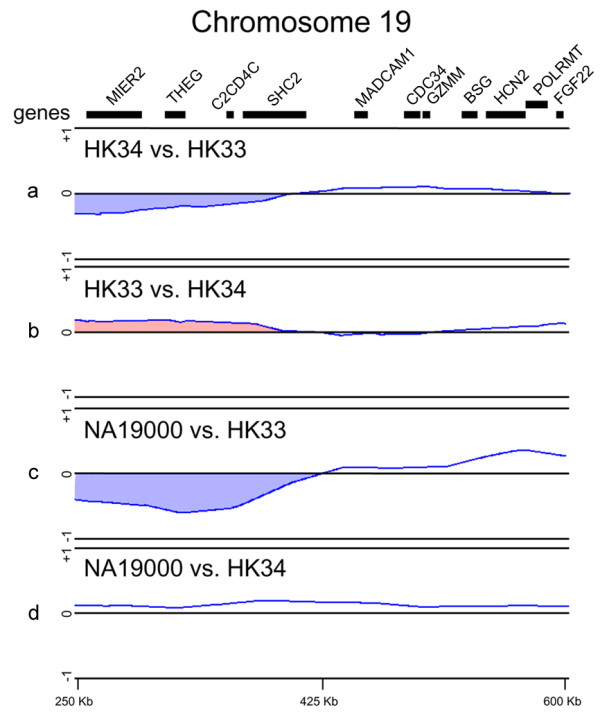
**High-density custom-made tiling microarray analysis of the MZ twins discordant for the MSA phenotype**. (a) Competitive hybridization of genomic DNA from the MSA-affected twin (HK33) versus that from his twin (HK34). (b) Dye-swap experiment of the normal twin (HK34) versus his affected twin (HK33). (c) Competitive hybridization of genomic DNA from the MSA-affected twin (HK33) versus that from a reference Japanese male (HapMap NA1900). (d) Competitive hybridization of genomic DNA from the MSA-affected twin (HK34) versus that from the reference Japanese male (HapMap NA19000). Each blue line represents a moving average ratio of log_2 _(Cy5/Cy3). The blue regions indicate deletions; these loci were defined by dye-swap experiments (red region). The top map shows the positions of putative genes in the region [16].

To validate the results of the CNV screening in both the affected MZ twin and 10 patients with MSA, we conducted high-density, custom-made oligonucleotide tiling microarray analysis. The DNAs from the twins were compared by competitive hybridization (Figure 3a), the hybridization results were verified by a dye-swap experiment (Figure 3b), and the DNAs were separately hybridized against a normal reference sample (HapMap NA1900; Figure 3c and 3d). We found heterozygous copy number loss (deletion) in the distal 350-kb region on the 19p subtelomere in the MSA-affected twin (HK33). The deleted region encompasses 4 genes including the (Src homology 2 domain containing)-transforming protein 2 (*SHC2*) gene on 19p13.3 (genomic positions 250-400).

### High-density tiling microarray analysis of the patients with MSA

**Figure 4:**
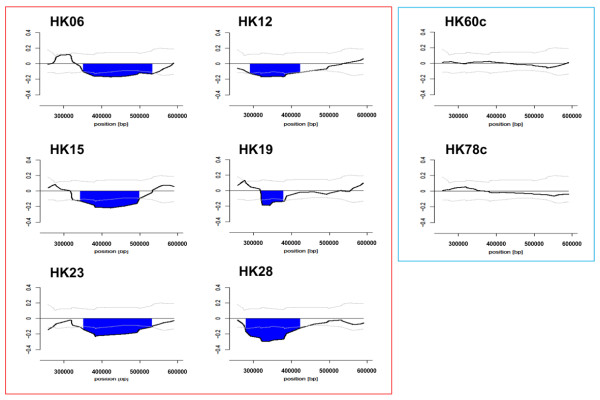
**Structure of copy number loss in the 350-kb subtelomeric region on 19p13.3 resolved by high-density tiling microarray**. The moving average log_2 _ratio (*y*-axis) is plotted against the genomic position along the chromosome (*x*-axis). HK06, HK12, HK15, HK19, HK23, and HK28 represent 6 patients with MSA and HK60c and HK78c represent controls. The dark lines indicate the copy number loss. The light lines and dotted lines indicate the normal range and median of the average log_2 _ratios for probes among normal individuals (*n *= 25), respectively.

**Figure 5:**
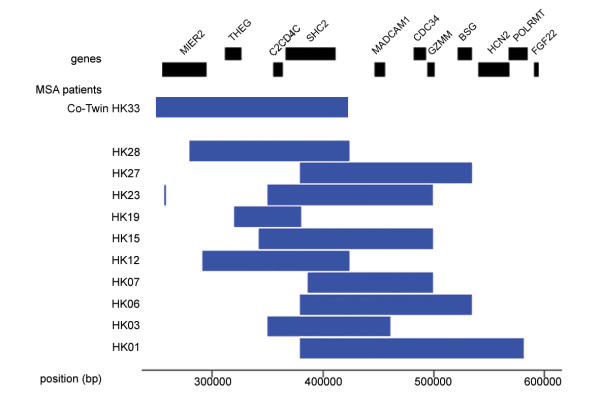
**Extent of copy number loss in the 350-kb subtelomeric region on 19p13.3 in the 10 patients with MSA**. The horizontal bars represent the length of the copy number loss region between genomic positions 250,000 (left) and 600,000 (right) in each patient. The extent of deletion observed in the affected MZ twin (HK33) is also shown. The top map shows the positions of putative genes in the region [16].

After confirming the absence of statistical differences in age and gender distribution, we further analyzed the distal 350-kb region showing copy number loss on the 19p subtelomere in the 33 patients with MSA against an additional set of 25 control subjects (second set of controls, Table 1) by using the same custom-made tiling array as for the MZ twins. Again, we found frequent heterozygous copy number loss in this region in 10 of the 33 patients but not in any of the 25 control subjects. Figure 4 demonstrates the moving average pattern of the distal 350-kb region with copy number loss on 19p13.3 in 6 of the 10 patients, and Figure 5 shows a deletion map of this region in the 10 patients.

Several genes are located in the subtelomeric region on 19p13.3, which were frequently deleted in the patients with MSA and the affected MZ twin (Figure 5). Of these genes, *SHC2*, which is expressed in the nervous system, is a prime candidate for MSA predisposition, because *SHC2 *impairment reportedly caused neurologic defects in a mouse model [17,18]. Therefore, heterozygous copy number loss of *SHC2 *would predispose individuals to MSA. Other genes in the region, such as hyperpolarization-activated cyclic nucleotide-gated potassium channel 2 (*HCN2*), mucosal vascular addressin cell adhesion molecule 1 (*MADCAM1*), and fibroblast growth factor 22 (*FGF22*) genes, may also have an etiologic link to MSA, because they exert their functions in the nervous system.

### Correlation between copy number loss of *SHC2 *and the MSA phenotype

Finally, we estimated the copy number loss of *SHC2 *in each patient and tested it for correlation with the phenotype at sampling (MSA-P vs. MSA-C) and at the onset of MSA (parkinsonism vs. cerebellar ataxia with/without autonomic failure) by chi-square test or Fisher's exact probability test. However, no significant correlations were noted (data not shown).

## Discussion

We employed 2 strategies for investigating MSA-specific CNVs: examination of de novo CNVs in a pair of MZ twins discordant for the MSA phenotype and CNV analysis of 33 patients with MSA, including the MZ twins. The CNV beadchip analysis enabled direct measurement of the fluorescent intensity of each marker, which is suitable for screening sporadic cases and control populations. The comparative genomic hybridization (CGH)-based whole-genome CNV oligonucleotide microarray enabled comparison of 2 similar genomes, which is ideal for detecting subtle differences between MZ twins. The combination of CGH-based genome-wide oligonucleotide CNV microarray and CNV beadchip analysis followed by region-targeting, high-density, custom-made oligonucleotide tiling microarray analysis led us to identify copy number loss of *SHC2 *and its neighboring genes in the 19p13.3 subtelomeric region of the affected MZ twin and 10 (30%) unrelated patients with MSA. Despite the limited number of subjects, this frequency is quite high when compared with those of other disorders: in schizophrenia, for instance, less than 1% in the patients has pathogenic CNV markers [13]. The copy number loss of *SHC2 *in the MSA-affected twin, which was validated by the population analysis, strongly suggests a causal link between genomic alteration and MSA phenotype.

Our results support the idea that CNV analysis of phenotypically discordant MZ twins is a powerful tool for identifying disease-predisposing loci. They also support the opinion that molecular analysis of phenotypically discordant MZ twins is an excellent focus for studying disease, because genotypic differences between twins derived from the same zygote highlight somatic variation [9]. Instances of somatic mosaicism by mutation in specific genes or chromosomal aberrations with links to disease have been described previously [19,20]. Our results also suggest that somatic mosaicism by pathogenic mutations affecting disease-susceptibility genes is observable frequently, rather than as an exception [9]. Further studies of a larger cohort of MZ twins discordant for MSA will be particularly useful for more detailed characterization of the genetic factors predisposing individuals to MSA.

We identified frequent heterozygous copy number loss of *SHC2 *and the surrounding area on the 19p13.3 subtelomeric region. This region contains a substantially larger number of low copy repeats and segmental duplications in a small area than that in the whole human genome. This complicated repetitive structure has prevented accurate mapping and sequencing analysis of the region [21]. The presence of numerous low copy repeats results in instability and can trigger frequent segmental loss by unequal crossover or end-joining events; therefore, such unstable CNV-rich subtelomeric regions are predisposed to deletion or duplication events [22]. Noteworthily, locus-specific mutation rates for CNVs or structural rearrangements are between 10^-6 ^and 10^-4^, at least 2 to 4 orders of magnitude (100- to 10,000-fold) greater than those for point mutations or SNPs [23]. Considering the causes of sporadic disease such as MSA, the new high mutation rate of CNVs is fascinating, and should be studied while searching for gene(s) related to such disorders [8]. CNVs in the distal 350-kb region of the 19p13.3 subtelomere have not been described for other neurologic diseases. For instance, our preliminary CNV beadchip analysis did not detect specific copy number loss in patients with multiple sclerosis (unpublished data).

*SHC2 *was deleted in the affected MZ twin and frequently deleted in the patients with MSA. *SHC2 *mRNA is expressed in various human adult tissues, including the nervous system. None of the CNVs around *SHC2 *described in the Database of Genomic Variants were associated with human neurologic disease [16]. In adult mice, Shc2 expression is limited to the nervous system, and in rat and mouse embryos, the expression of *Shc2 *mRNA is the highest in the dorsal root ganglia and superior cervical ganglia [17,18]. Shc proteins act as molecular switches in neuronal cell development from proliferation to survival and/or differentiation.

Several other genes frequently deleted in patients with MSA, such as *HCN2*, *MADCAM1*, and *FGF22*, are also expressed in nervous tissue. *HCN2 *contributes to spontaneous rhythmic activity in the external segment of the globus pallidus [24,25]. Augmented currents through the channel of an *HCN2 *variant was described in patients with febrile seizure syndrome [26]. Human *MADCAM-1 *mRNA transcripts are expressed in the brain and abnormal elevation of *MADCAM-1 *has been described in patients with multiple sclerosis [27]. *FGF22 *plays an essential role in nervous system development and is expressed by cerebellar granule cells [28]. Copy number loss of these genes may alter their expression or produce aberrant or unstable mRNA or protein products. Furthermore, CNVs influence the expression of not only the genes harboring them but also the genes in their vicinity extending up to half a megabase [29]. The mechanism of the influence of CNVs on the phenotype over multiple gene expressions is yet to be studied.

Specific copy number loss indicates the possibility that MSA is a genomic disorder derived from genetic hotspots harboring unstable structures predisposed to CNVs. In this regard, rare clustering of MSA within families could provide information on inherited genetic variants related to the development of this disorder [30].

## Conclusions

Our study provides compelling evidence of heterozygous copy number loss in the *SHC2 *region in the affected MZ twin and one-third of the patients with MSA. Further studies on the function of *SHC2 *in the nervous system help to elucidate the pathogenesis of MSA and identify novel therapeutic targets for the disease. Studies of independent populations with MSA and different ethnicities are warranted to fully understand the etiology of MSA, including yet unidentified genetic and/or nongenetic factors.

## List of abbreviations

BSG: basigin; CDC34: cell division cycle 34 homolog (*S. cerevisiae*); CNV: copy number variation; CGH: comparative genomic hybridization; C2CD4C: C2 calcium-dependent domain containing 4C; FGF22: fibroblast growth factor 22; GCI: glial cytoplasmic inclusion; GZMM: granzyme M; HCN2: hyperpolarization-activated cyclic nucleotide-gated potassium channel 2; MADCAM1: mucosal vascular addressin cell adhesion molecule 1; MIER2: mesoderm induction early response 1, family member 2; MRI: magnetic resonance imaging; MSA: multiple system atrophy; MZ: monozygotic; POLRMT: polymerase (RNA) mitochondrial (DNA directed); SHC2: (Src homology 2 domain containing)-transforming protein 2; SNP: single-nucleotide polymorphism; THEG: testicular haploid expressed gene homolog (mouse)

## Competing interests

The authors declare that they have no competing interests.

## Authors' contributions

H. Sasaki and ME conceived the study, conducted the molecular genetic studies and data interpretation, had full access to the data, accept responsibility for the integrity of the work and accuracy of the data analysis, and drafted the manuscript as well as provided administrative, technical, and material support. HI conducted the molecular genetic studies and data interpretation, participated in designing the study, and performed the statistical analysis. NI and H. Sato conducted the molecular genetic studies and data interpretation. IY participated in designing the study and performed the statistical analysis as well as provided administrative, technical, and material support. TK conducted the molecular genetic studies and data interpretation as well as provided administrative, technical, and material support. KM and JU participated in supervising the study and critically revising the manuscript content. All authors read and approved the final manuscript.

## Supplementary Material

Additional file 1 **Figure S1 - Pattern of the CNV region on 2p25.3 in the MZ twins discordant for the MSA phenotype by CGH-based whole-genome CNV microarray analysis**. (Top panel) Competitive hybridization of genomic DNA from the MSA-affected twin (HK33) versus that from his twin (HK34). (Bottom panel) Dye-swap experiment of the normal twin (HK34) versus his affected twin (HK33). Each blue line represents a moving average ratio of log_2 _(Cy5/Cy3). The blue region indicates deletion. These loci were defined by dye-swap experiments (red region). Click here for file

Additional file 2 **Figure S2 - Pattern of the CNV region on 4q35.2 in the MZ twins discordant for the MSA phenotype by CGH-based whole-genome CNV microarray analysis**. (Top panel) Competitive hybridization of genomic DNA from the MSA-affected twin (HK33) versus that from his twin (HK34). (Bottom panel) Dye-swap experiment of the normal twin (HK34) versus his affected twin (HK33). Each blue line represents a moving average ratio of log_2 _(Cy5/Cy3). The blue region indicates deletion. These loci were defined by dye-swap experiments (red region). Click here for file

Additional file 3 **Figure S3 - Pattern of the CNV region on 19p13.3 in the MZ twins discordant for the MSA phenotype by CGH-based whole-genome CNV microarray analysis**. (Top panel) Competitive hybridization of genomic DNA from the MSA-affected twin (HK33) versus that from his twin (HK34). (Bottom panel) Dye-swap experiment of the normal twin (HK34) versus his affected twin (HK33). Each blue line represents a moving average ratio of log_2 _(Cy5/Cy3). The blue region indicates deletion. These loci were defined by dye-swap experiments (red region). Click here for file

Additional file 4 **Figure S4 - Pattern of the CNV region on 2p25.3 in the patients with MSA and controls**. Data measured by CNV 57K beadchip analysis were analyzed by the Hidden Malcov Model. The genomic structures of 100 normal control subjects (top) and 33 patients with MSA (bottom) are horizontally aligned from position 000,000 (left) to position 5,000,000 (right). Each blue square represents copy number loss at each CNV probe site whereas each red square represents copy number gain. Click here for file

Additional file 5 **Figure S5 - Pattern of the CNV region on 4q35.2 in the patients with MSA and controls**. Data measured by CNV 57K beadchip analysis were analyzed by the Hidden Malcov Model. The genomic structures of 100 normal control subjects (top) and 33 patients with MSA (bottom) are horizontally aligned from position 186,000,000 (left) to position 191,000,000 (right). Each blue square represents copy number loss at each CNV probe site whereas each red square represents copy number gain. Click here for file
