# Supplementary figures and images for: Copy number loss of (src homology 2 domain containing)-transforming protein 2 (SHC2) gene: discordant loss in monozygotic twins and frequent loss in patients with multiple system atrophy
Source: Mol Brain. 2011 Jun 10;4:24. doi: 10.1186/1756-6606-4-24 (PMC3141657; doi:10.1186/1756-6606-4-24)

# Chromosome 2

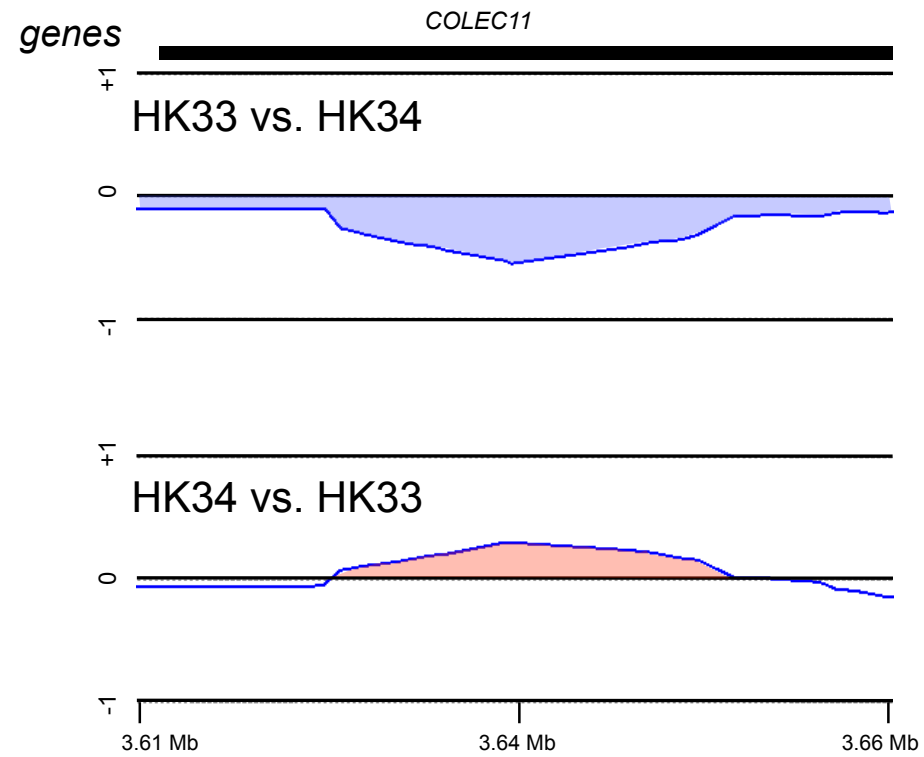

Supplement: Additional file 1 — Figure S1 - Pattern of the CNV region on 2p25.3 in the MZ twins discordant for the MSA phenotype by CGH-based whole-genome CNV microarray analysis. (Top panel) Competitive hybridization of genomic DNA from the MSA-affected twin (HK33) versus that from his twin (HK34). (Bottom panel) Dye-swap experiment of the normal twin (HK34) versus his affected twin (HK33). Each blue line represents a moving average ratio of log2 (Cy5/Cy3). The blue region indicates deletion. These loci were defined by dye-swap experiments (red region). [file 1756-6606-4-24-S1.PDF]

# Chromosome 4

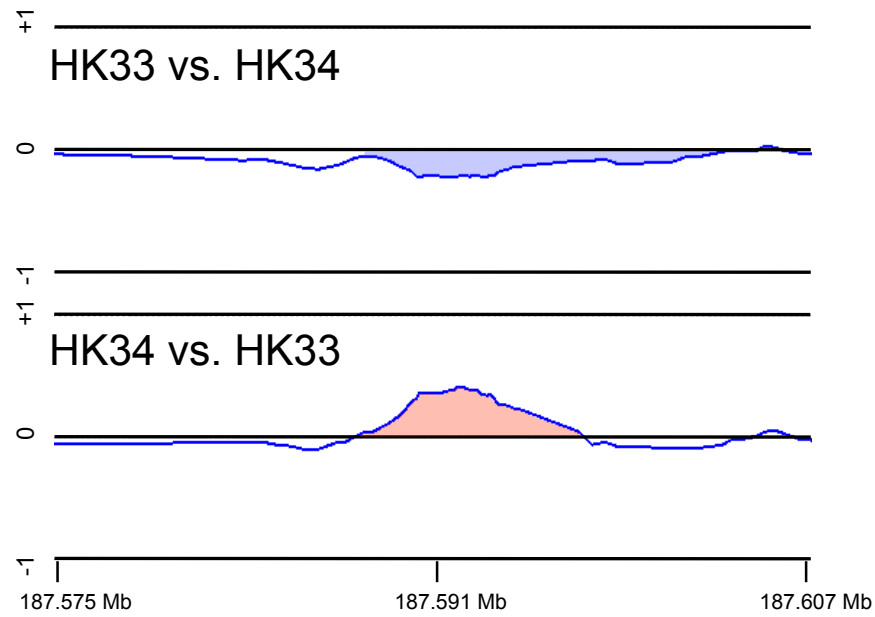

Supplement: Additional file 2 — Figure S2 - Pattern of the CNV region on 4q35.2 in the MZ twins discordant for the MSA phenotype by CGH-based whole-genome CNV microarray analysis. (Top panel) Competitive hybridization of genomic DNA from the MSA-affected twin (HK33) versus that from his twin (HK34). (Bottom panel) Dye-swap experiment of the normal twin (HK34) versus his affected twin (HK33). Each blue line represents a moving average ratio of log2 (Cy5/Cy3). The blue region indicates deletion. These loci were defined by dye-swap experiments (red region). [file 1756-6606-4-24-S2.PDF]

# Chromosome 19

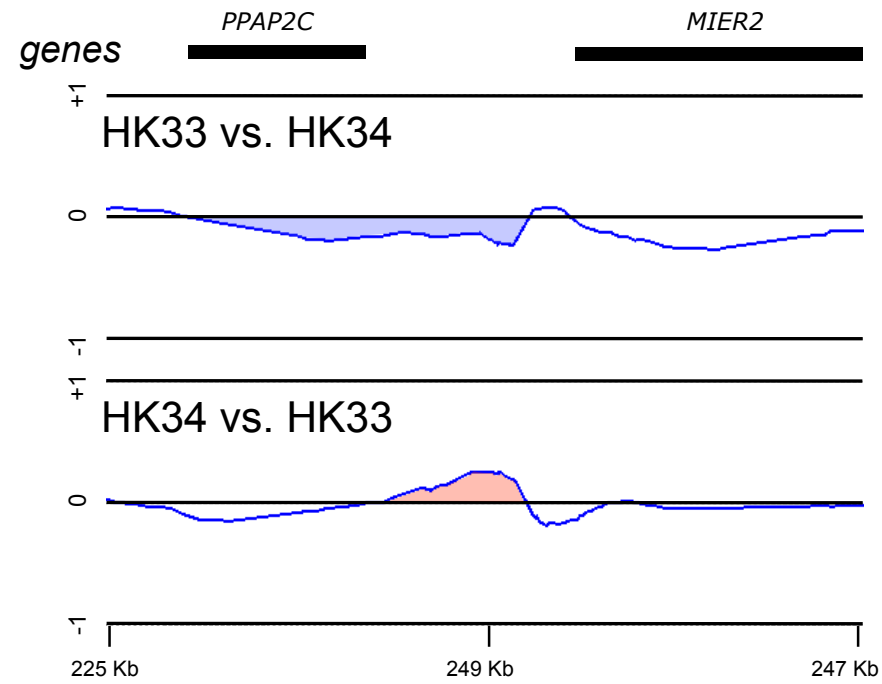

Supplement: Additional file 3 — Figure S3 - Pattern of the CNV region on 19p13.3 in the MZ twins discordant for the MSA phenotype by CGH-based whole-genome CNV microarray analysis. (Top panel) Competitive hybridization of genomic DNA from the MSA-affected twin (HK33) versus that from his twin (HK34). (Bottom panel) Dye-swap experiment of the normal twin (HK34) versus his affected twin (HK33). Each blue line represents a moving average ratio of log2 (Cy5/Cy3). The blue region indicates deletion. These loci were defined by dye-swap experiments (red region). [file 1756-6606-4-24-S3.PDF]

Normal(n=100)

MSA(n=33)

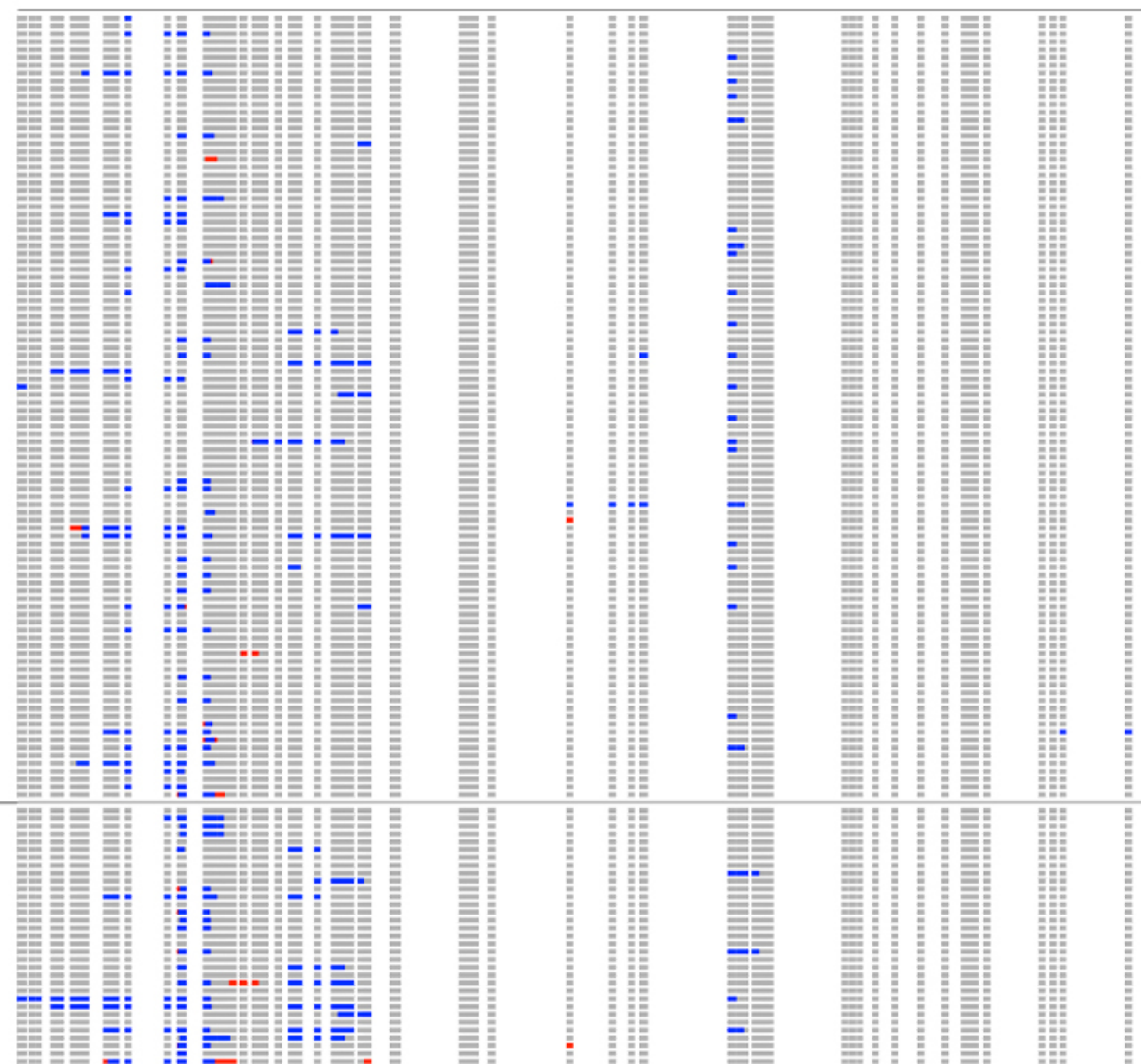

0 1M 2M 3M 4M 5M (bp)

Chr2

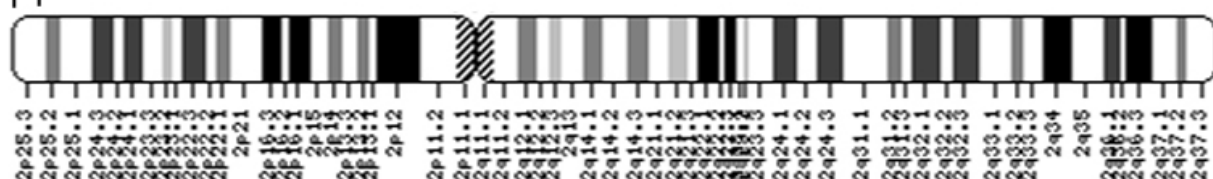

Supplement: Additional file 4 — Figure S4 - Pattern of the CNV region on 2p25.3 in the patients with MSA and controls. Data measured by CNV 57K beadchip analysis were analyzed by the Hidden Malcov Model. The genomic structures of 100 normal control subjects (top) and 33 patients with MSA (bottom) are horizontally aligned from position 000,000 (left) to position 5,000,000 (right). Each blue square represents copy number loss at each CNV probe site whereas each red square represents copy number gain. [file 1756-6606-4-24-S4.PDF]

Normal(n=100)

MSA(n=33)

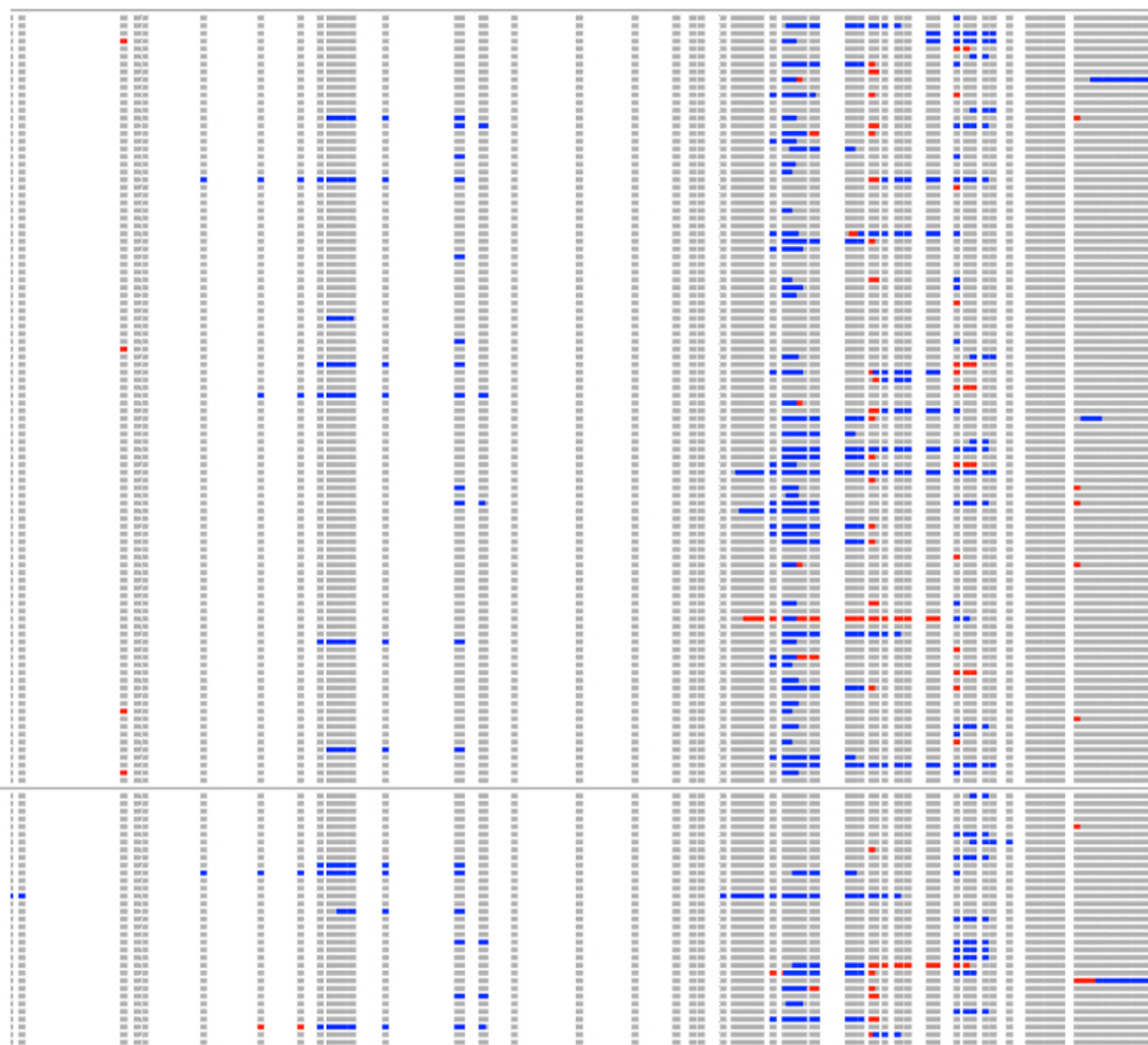

187M

188M

189M

190M

191M (bp)

Chr4

4p16.3  
4p16.2  
4p16.1  
4p15.3  
4p15.32  
4p15.31  
4p15.2  
4p15.1  
4p14  
4p13  
4p12  
4p11  
4q12  
4q13.1  
4q13.2  
4q13.3  
4q21.1  
4q21.2  
4q21.3  
4q21.31  
4q22.1  
4q22.2  
4q22.3  
4q23  
4q24  
4q25  
4q26  
4q27  
4q28.1  
4q28.2  
4q29.3  
4q31.1  
4q31.2  
4q31.3  
4q31.4  
4q32.1  
4q32.2  
4q32.3  
4q33  
4q34.1  
4q34.2  
4q34.3  
4q35.1  
4q35.2

Supplement: Additional file 5 — Figure S5 - Pattern of the CNV region on 4q35.2 in the patients with MSA and controls. Data measured by CNV 57K beadchip analysis were analyzed by the Hidden Malcov Model. The genomic structures of 100 normal control subjects (top) and 33 patients with MSA (bottom) are horizontally aligned from position 186,000,000 (left) to position 191,000,000 (right). Each blue square represents copy number loss at each CNV probe site whereas each red square represents copy number gain. [file 1756-6606-4-24-S5.PDF]
